# An Adoptive Threshold-Based Multi-Level Deep Convolutional Neural Network for Glaucoma Eye Disease Detection and Classification

**DOI:** 10.3390/diagnostics10080602

**Published:** 2020-08-18

**Authors:** Muhammad Aamir, Muhammad Irfan, Tariq Ali, Ghulam Ali, Ahmad Shaf, Alqahtani Saeed S, Ali Al-Beshri, Tariq Alasbali, Mater H. Mahnashi

**Affiliations:** 1 Department of Computer Science, COMSATS University Islamabad Sahiwal Campus, Sahiwal 57000, Pakistan; muhammadaamir@cuisahiwal.edu.pk (M.A.); ahmadshaf@cuisahiwal.edu.pk (A.S.); 2 College of Engineering, Electrical Engineering Department, Najran University, Najran 61441, Saudi Arabia; 3 Department of Computer Science, University of Okara, Okara 56300, Pakistan; ghulamali@uo.edu.pk; 4 Department of Surgery, Faculty of Medicine, Najran University, Najran 61441, Saudi Arabia; alhafezsaeed@gmail.com; 5 King Khalid Eye Specialist Hospital, Riyadh 12329, Saudi Arabia; as.beshri@gmail.com; 6 Department of Ophthalmology, College of Medicine, Al Imam Mohammad Ibn Saud Islamic University (IMSIU), Riyadh 4233-13317, Saudi Arabia; taalasbali@imamu.edu.sa; 7 Department of Pharmaceutical Chemistry, College of Pharmacy, Najran University, Najran 61441, Saudi Arabia; matermaha@gmail.com

**Keywords:** ML-DCNN, glaucoma deep-learning, computer vision, convolutional neural network, glaucoma eye disease

## Abstract

Glaucoma, an eye disease, occurs due to Retinal damages and it is an ordinary cause of blindness. Most of the available examining procedures are too long and require manual instructions to use them. In this work, we proposed a multi-level deep convolutional neural network (ML-DCNN) architecture on retinal fundus images to diagnose glaucoma. We collected a retinal fundus images database from the local hospital. The fundus images are pre-processed by an adaptive histogram equalizer to reduce the noise of images. The ML-DCNN architecture is used for features extraction and classification into two phases, one for glaucoma detection known as detection-net and the second one is classification-net used for classification of affected retinal glaucoma images into three different categories: Advanced, Moderate and Early. The proposed model is tested on 1338 retinal glaucoma images and performance is measured in the form of different statistical terms known as sensitivity (SE), specificity (SP), accuracy (ACC), and precision (PRE). On average, SE of 97.04%, SP of 98.99%, ACC of 99.39%, and PRC of 98.2% are achieved. The obtained outcomes are comparable to the state-of-the-art systems and achieved competitive results to solve the glaucoma eye disease problems for complex glaucoma eye disease cases.

## 1. Introduction

Eyes are the most used sensory organ of the human body among the five senses. A significant segment of the mind is utilized in visual processing. Glaucoma, usually caused by increased pressure inside the eye, is the primary root of visual loss over the globe and cannot be rehabilitated. Detection of glaucoma in its beginning is difficult but can be cured [1]. Glaucoma analysis is based on the medicinal history of the patient’s family, intraocular pressure (IOP), retinal nerve fiber layer thickness, and changes in optic disc (OD) structure, for example, the distance across, volume, and region. According to a study, in 2013 overall 64.3 million people in the population aged 40 to 80 years experienced glaucoma. This number can be exceeded to 76 million by 2020 and 111.8 million by 2040 [2].

The retina layer is composed of roughly one million nerve fibers that organize collectively to make the optic nerves. The start of the optic nerves within the retina layer is termed as an optic disk (OD) or optic nerve head (ONH), which is circular in form and noticeably intense within the retinal images. Symptoms of glaucoma only occur when the disease is slightly advanced; glaucoma is called the silent thief of sight. Therefore, the timely diagnosis of this disease is necessary [3]. Eye screening is a too long and tiresome process because of the keen checkup of each individual patient. To improve the eye screening procedure, a computer aided diagnosis system (CADx) can be used to give more productive results to the patients to distinguish between healthy and infected retinal fundus images as it is hard for oculists to label this distinction accurately.

Over the most recent couple of years, deep learning algorithms have reformed the field of computer vision and are now becoming part of our everyday lives [4]. The machine learning algorithms are appropriate for the analysis of glaucoma. Generally, two primary methodologies utilized for glaucoma identification are segmentation-based and features learning-based approaches. The digital fundus images are utilized for the recognition of glaucoma.

In previous works, the researchers provided a solution for automatic detection and classification of glaucoma via segmentation of the cup to extract the features [5]. To segment the optic disk (OD) and optic cup regions in a sturdy way is a troublesome assignment for the computer-aided system. It requires lots of image processing methods and domain expert knowledge to pick the most biased features. Diagnosing methods on eye fundus images are based on the segmentation of blood vessels and the optic disc regions. Any destruction to the retinal nerve fiber layer (RNFL) is distinguished by utilizing the texture features of retinal fundus images [6].

The objective of this work is to give an automated framework for glaucoma detection through retinal image analysis, which contains phases: a collection of a retinal image database, preprocessing to minimize the amount of noise existing in images, features training, and finally the classification of images as glaucomatous or not. The convolutional neural network (CNN) architecture will be responsible for features learning. The terms accuracy, specificity, sensitivity, and receiver operating characteristic/area under curve (ROC/AUC) have been commonly used as a benchmark for evaluating a diagnosis system. Evaluation of our proposed framework will be performed by using a database containing retinal fundus images of patients from the local hospital.

## 2. Materials

Machine-learning algorithms are appropriate for various complicated image classification problems such as glaucoma disease classification from retinal images [7,8,9]. Glaucoma is a chronic eye disease caused by eye retinal changes [10] which leads to gradual vision loss and, finally, complete blindness occurs if not diagnosed timely [11,12]. Glaucoma-Deep, a feature-based learning framework was proposed which contains four phases: identification, extraction, optimization, and classification. They utilized a convolutional neural network (CNN) to separate features and supervised a deep-belief network (DBN) to enhance the extracted features and a linear SoftMax classifier to classify amongst ordinary and glaucoma eyes. In [13] tested the C5.0, k-nearest neighbor (kNN), support vector machine (SVM), and random forest (RF) learning algorithms for glaucoma identification in the light of retinal nerve fiber layer (RNFL) density and visual field. Among all, the RF model provided high-quality performance with an accuracy of 0.98%: a computerized system for glaucoma identification.

In [14] tested 702 retinal images. Initially, RGB images transform to grayscale images by the luminance technique. They utilized a local configuration pattern (LCP) for features extraction, sequential floating forward search (SFFS) for features selection, *t*-test for ranking, and k-nearest neighbor (kNN) classifier for classification. In [15], two datasets, public and private, were used. A 2D Empirical Wavelet Transform (EWT) was utilized to breakdown fundus images and acquire features from decomposed EWT for glaucoma identification. Those features were graded via a *t*-test by using a classifier Least-Squares SVM.

In [16], authors covered the segmentation and localization of the optic disc head diagnosis using 3-D datasets, pixel-level glaucomatous changes, and the artificial neural network (ANN) for recognizing the continuation of glaucoma disorder. In [17], a multilayer convolutional neural network deployed and split into four convo layers adjacent to two fully connected layers for glaucoma detection. In [18], RNFL and optic nerve data utilized to test the performance of machine-learning classifiers and RF. The system obtains a 0.877 value of region under the receiver operating characteristic (ROC) via RF. Glaucoma was identification using SVM rather than using a deep-learning algorithm, and separated color, texture features from fundus images [19].

The system was tested on 100 people and got a specificity of 87% and sensitivity of 100%. Ref. [20] applied feature-based learning on fundus images for glaucoma and choose CNN for feature-learning with an activation function. They utilized normal and glaucomatous eye patterns to evolve for the schooling of Convolutional neural architecture. ORIGA and SCES datasets were used with the area under curve (AUC) values of 0.838 and 0.898, distinctively, to introduce a model to section optic-disc features extraction in a distinct color style and categorize them by a multilayer perceptron (MLP) framework. Automatic recognition of the eye disorder with image processing techniques was performed through machine-learning classifiers [21].

In [22], a dataset of 1542 fundus images was used, including 786 healthy and 756 glaucoma patients. All these pictures were settled into a 1× (240 × 240 × 3) one-dimensional array to perform logistic regression. The Google Net Inception v3 model with a modified classification layer was used to fulfill their classification needs. They have used an Adam optimizer as an optimization function for backpropagation, and cross-entropy as a loss function. Performance of the developed model and Google Net model depended on the ROC curve by computing specificity and sensitivity of the models. The final version accomplished accuracy and AUC on the test data, training data, and validation data were 87.9% and 0.94%, 92.2% and 0.98%, and 88.6% and 0.95%, respectively.

In [23], the presented design resembles the original U-Net; it comprises a contracting (left aspect) path and an expansive (right aspect) path. The contracting way basically rehashes the commonplace engineering of the convolutional part of the classification organizer. On the expansive way, data are converged from layers of the contracting way of suitable goals and layers of broadway of lower goals. Results are reported for freely accessible datasets Drions-db, Rim-One, and Drishti. Analyses results and a visual correlation demonstrate that programmed optic disc division should be possible at the quality aggressive with humans.

In [24] carries out experiments with convolutional neuronal networks to achieve the automatic detection of this disease. For this purpose, they have collected 25 fundus images of normal eye and 19 fundus images of glaucoma patients and then re-trained the Inception v3 convolutional neuronal network. The trained model provides on average a 99% accuracy for classifying glaucoma and no glaucoma. They still believe that a better predictive model could be generated by retraining the algorithm using more fundus images.

## 3. Methodology

The methodology section defines the overall methodology of the proposed Multi-Level Deep Convolutional Neural Network (ML-DCNN) for glaucoma eye disease detection and classification. The current machine learning and artificial intelligence methods for Glaucoma eye detection have the least number of filters and large time complications. To tackle this issue, Multi-Level Deep Neural Network-based image classification has been proposed. In our work, we chose a features-based learning approach to the multilayer deep Convolutional Neural Network (CNN). We deal with datasets of retinal fundus images. In the proposed research, a Multi-Level Deep Convolutional Neural Network (ML-DCNN) is proposed and implemented for glaucoma eye disease detection and classification. It consists of different CNN layers. It is implemented in two phases: the first one is Detection-Net CNN (DN-CNN) and the second one is Classification-Net (CN-DCNN) with different layers as their detail explained in Algorithm 1.

### 3.1. Multi-Level Deep CNN (ML-DCNN) Architecture

**Figure 1:**
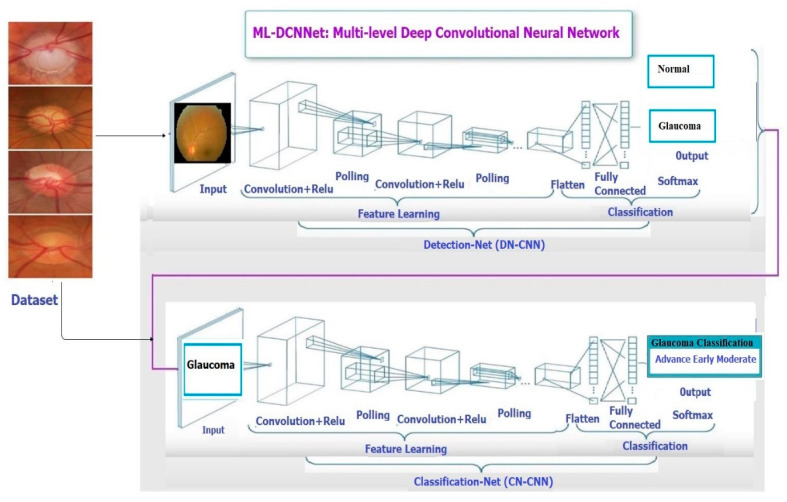
The Architecture of Multi-Level deep Convolutional Neural Network (ML-DCNN).

**Figure 2:**
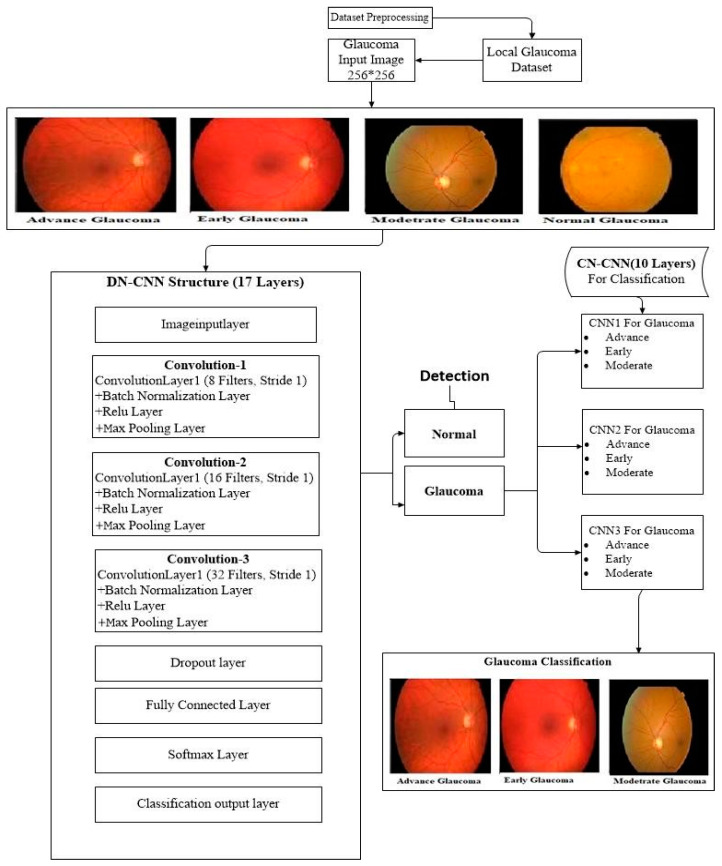
The Representation of Working Process of the (ML-DCNN).

A Multi-Level Deep Convolutional Neural Network is introduced. It is related to an ordinary neural network having a combination of different neuron layers, learning rates, and other parameters. In this research for the selection of networks, we used four different CNN approaches as shown in Figure 1. The working process of the proposed Multi-Level Deep Convolutional Neural Network is represented in Figure 2. All the CNNs were implemented in two parts as explained below.
**Algorithm 1** Proposed Glaucoma detection and classification framework.**Input:** Image sequences **x** with three class labels where **x ∈ t** (where **t** = 1,2,3).**Outputs:** Predicted glaucoma detection for each image sequence and classification for each image.   1. Divide CNN network into two parts: Detection-Net CNN for glaucoma detection and Classification-Net CNN for glaucoma classification estimation.   2. Partition data into training and test sets.   3. Form a pool of apex features based on the training set for Detection-Net.   4. Part 1: Detection-Net   5. **if** detect Normal   6. **stop**   7. **else** glaucoma   8. **end if**   9. **Part 2:** Classification-Net   10. **for** each glaucoma image sequence **do**   11. **for**
*x* = 1 to *t*
**do**   12. Train a Classification-Net CNN for each glaucoma class.   13. **end for**   14. **end for**   15. **for** each glaucoma test sequence **do**   16. Obtain predicted glaucoma disease levels (Advance, Early, and Moderate).   17. Construct an array of glaucoma disease levels.   18. **end for**

### 3.2. Detection-Net CNN (DN-CNN)

**Figure 3:**
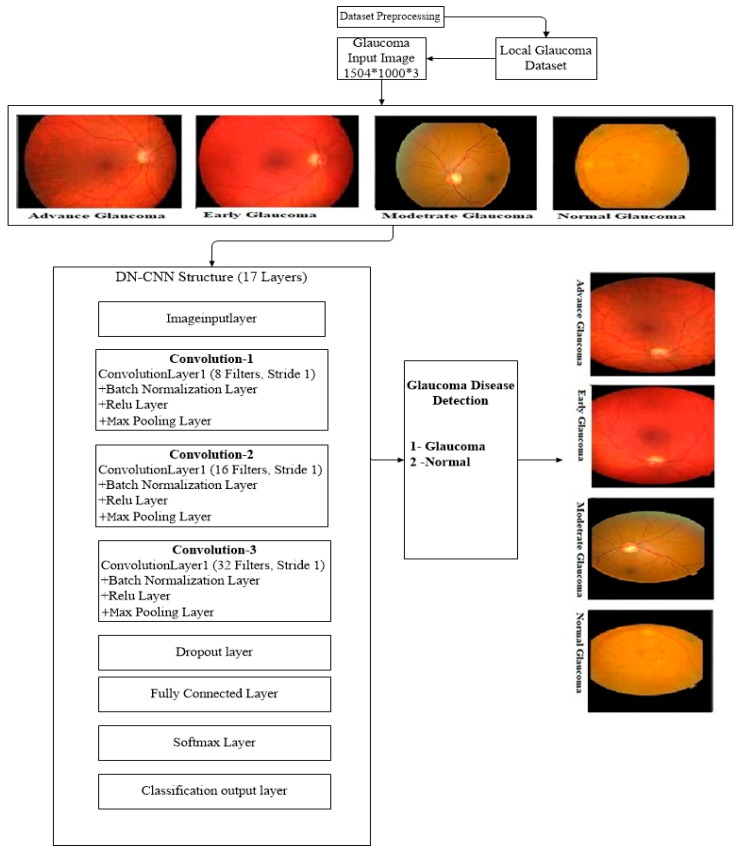
Layer-Wise Representation of the Architecture of Detection-Net-CNN.

In this portion, the first CNN is used to detect glaucoma disease. It is known as Detection-Net (DN-CNN) as shown in Figure 3. In this Net, we used a CNN having 17 layers: an image input layer, 3 convolutional layers, 3 batch normalization Layer, 3 ReLu layers, 3 max-pooling layers, a dropout layer, and fully connected SoftMax and classification layers as shown in Table 1. In this table the input image has initially a 256 × 256 size with a filter size 3 × 3, the number of filters (k) 8, and a stride 1 for the first convolutional layer. In the second convolutional layer, the size decreases to 128 × 128; filter size, and stride remains the same with 16 number of filters. In the third convolutional layer, the size is 64 × 64 with 32 filters and filter size and stride remain the same. This network, first of all, takes an image as an input in the image input layer and passes through the different CNN layers. The image passes through all the CNN layers to get the features matrix of the image, and at the end of the network classification layer produces the corresponding classification label. This classification is further used as an input in the next classification network to estimate the glaucoma disease level (Advance, Moderate, Early).

### 3.3. Classification-Net CNN (CN-CNN)

**Figure 4:**
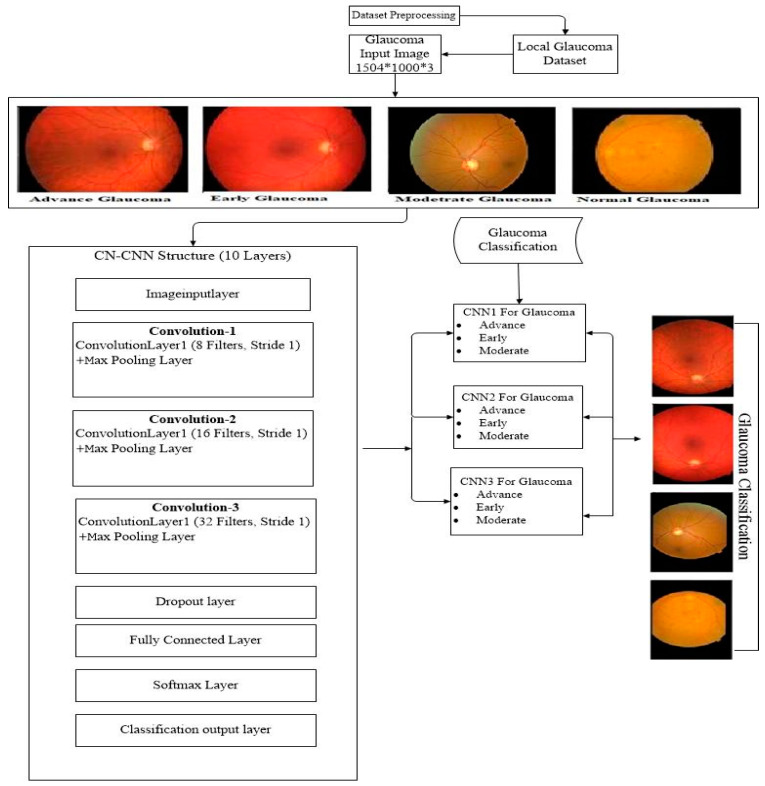
Layer-Wise Representation of the Architecture of Classification-Net-CNN.

In this part of ML-DCNN architecture, four CNNs are used for glaucoma disease level classification. It is named as Classification-Net CNN (CN-CNN). This network uses classified images from the DN-CNN network to detect glaucoma affected images. The disease level of glaucoma is divided into four phases: advance, early, moderate, and normal, where early describes the beginning of the glaucoma disease, moderate belongs to medium value, advance defines the peak value, and normal explains the no glaucoma disease value. For each stage in the glaucoma detection in the Classification-Net phase, we implemented one CNN algorithm, so a total of four CNN architectures are used in this part. The architecture of CN-CNN is shown in Figure 4; we used 10 layers with a learning rate of 0.001 and 30 epoch size as explained in Table 2. In this table, the input image has initially a 256 × 256 size with a filter size 3 × 3; the number of filters (k) is 8, and stride is 1 for the first convolutional layer. In the second convolutional layer, the size decreases to 126 × 126; filter size, and stride remains the same with 16 filters. In the third convolutional layer, the size is 61 × 61 with 32 filters and filter size and stride remains the same.

## 4. Results and Discussion

The proposed Multi-Level Deep Convolutional Neural Network (ML-DCNN) was tested on the personal laptop with an Intel Core i7, 2.60 GHz CPU with 8GB RAM in the MATLAB R2018a and different resultant statistical values were calculated.

### Dataset Pre-Processing

**Figure 5:**
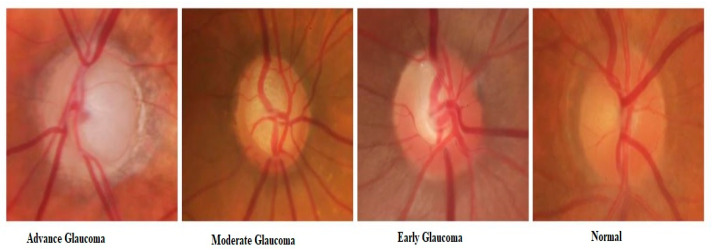
Glaucoma Stages.

The local retinal glaucoma image dataset is pre-processed by an adaptive histogram equalizer to decrease the amount of noise existing in the images. The local retinal glaucoma dataset images are acquired from different private and public resources in different hospitals. The dataset consists of a total of 1338 retinal images. Each image belongs to one of four classes i.e., Normal, Early Glaucoma, Moderate Glaucoma, Advanced Glaucoma. The dataset is a kind of imbalanced dataset because there are about 79% of images that belong to one class and the remaining 21% of images belong to the remaining three classes. There are 8.96% early glaucoma images, 5.98% moderate glaucoma images, 5.98% advanced glaucoma images, and 79.08% normal images (with no glaucoma). The detail of the dataset and its distribution into training, validation, and testing subsets has been shown in Table 3. To do training and testing on the given dataset, the dataset of 803 images is divided into four categories as advance, early, moderate, and normal with 50, 75, 50, and 628 images, respectively, as shown in the third column of Table 4. The dataset is divided into test, training, and validation datasets. The ratio of testing, validation, and training dataset is 23%, 17%, and 60%, respectively, as shown in Table 4. Three expert clinical assistants were requested to make a difference between four stages of glaucoma eye disease as shown in Figure 5. The majority of voting was used to assign labels to the images where experts disagreed.

The results of the proposed ML-DCNN are calculated with the help of the following statistical equations.

Sensitivity (SE) =TP/(TP+FN)(1)

Specificity (SP) =TN/(TP+FP)(2)

Accuracy (ACC) = (TP+TN)/(TP+FN+TN+FP)(3)

Precision (PRC) =TP/(TP+FP)(4)

**Figure 6:**
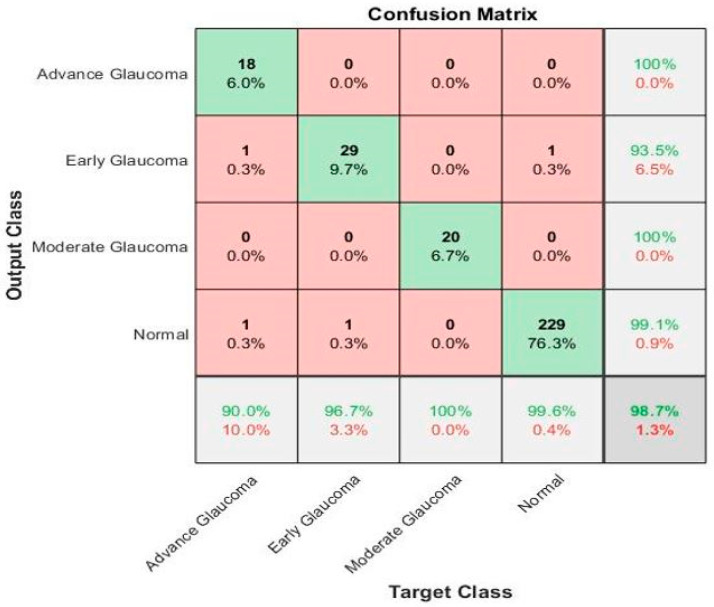
Confusion matrix of the 4-class glaucoma classification using the proposed ML-DCNN model on the Testing Dataset.

Here, TP denotes true positive that is correctly identified between glaucoma images, and TN explains the true negative that identified wrongly classified images. Whereas, false positive (FP) and false-negative (FN) denotes the correctly and wrongly identified classes, as shown in Figure 6.

The performance of these parametric equations is calculated on 300 test retinal glaucoma disease images based on a ground truth and proposed ML-DCNN model, as shown in Table 5 and Figure 6.

The confusion matrix in Figure 6 shows the results of the recognition rate of different classes on the testing dataset. The rows show the predicted values of the classes and the columns explained the true class values. The diagonal cells show the total number of observations that are correctly classified. The off-diagonal cells explain the incorrect classification of observations. Each cell includes the total number of observations and their percentage. The predicted values’ percentage that is correctly and incorrectly classified for each class is presented in the far-right column of the confusion matrix. These values are also called the precision (positive predictive value) and false discovery rate, correspondingly. The correct and incorrect classification percentages of all the classes are explained in the row at the bottom end. These values are frequently called the recall (or true positive rate) and false negative rate, correspondingly. The overall accuracy is shown in the bottom-right cell of the confusion matrix.

**Figure 7:**
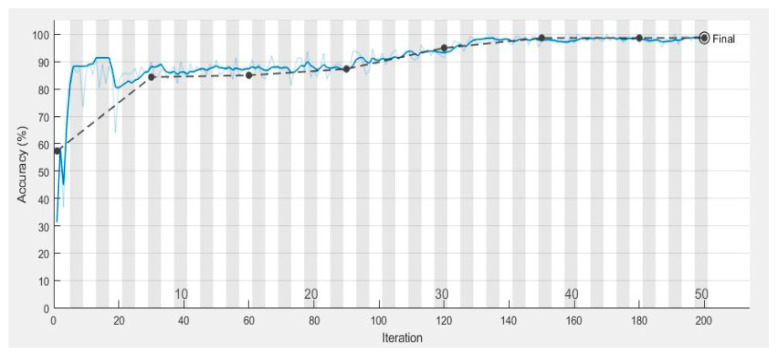
Graphical Representation of Validation and Training Accuracy over the various number of iterations.

**Figure 8:**
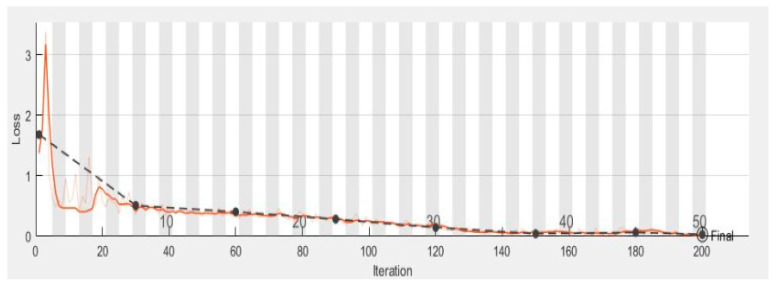
Graphical Representation of Validation and Training Loss Function over the various number of iterations.

Figure 7 shows the validation and training accuracy which is 98.7%, and Figure 8 shows the validation and training loss. Table 4 shows a comparison of the performance of the proposed ML-DCNN algorithm. It shows the performance in the form of an average SE 98.16%, SP 99.35%, ACC 99.28%, and PRC 96.41%. The proposed ML-DCNN glaucoma model obtained different statistical values for advanced glaucoma, early glaucoma, moderate glaucoma, and normal glaucoma categories as shown in Table 4. The obtained outcomes are comparable to the state-of-the-art systems and achieved competitive results to solve the glaucoma eye disease problems for complex glaucoma eye disease cases.

**Figure 9:**
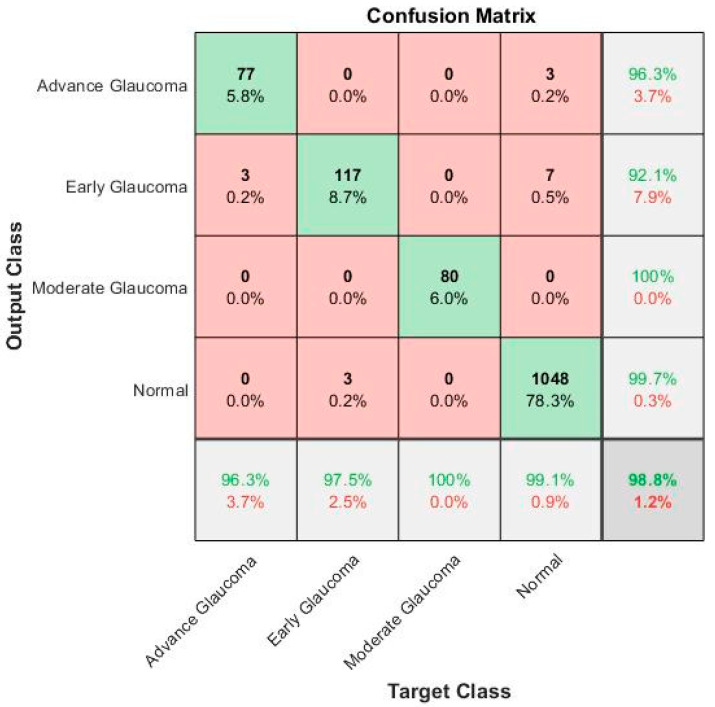
Confusion matrix of the 4-class glaucoma classification using the proposed ML-DCNN Model on the Local Retinal Glaucoma Image Dataset.

Next, we evaluated our proposed framework on the complete dataset of glaucoma images. The confusion matrix in Figure 9 shows the results of the recognition rate of different classes on the local retinal glaucoma image dataset. The rows show the predicted values of the classes and the columns explained the true class values. The diagonal cells show the total number of observations that are correctly classified. The off-diagonal cells explain the incorrect classification of observations. Each cell includes the total number of observations and their percentage. The predicted values’ percentages that are correctly and incorrectly classified for each class are presented in the far-right column of the confusion matrix.

These values are also called the precision (positive predictive value) and false discovery rate, correspondingly. The correct and incorrect classification percentages of all the classes are explained in the row at the bottom end. These values are frequently called the recall (or true positive rate) and the false negative rate, correspondingly. The overall accuracy is shown in the bottom-right cell of the confusion matrix.

Table 5 shows the performance evaluation of the proposed ML-DCNN model on the local retinal glaucoma image dataset. It shows the performance in the form of an average SE 97.04%, SP 98.99%, ACC 99.39%, and PRC 98.2% is observed on a complete dataset. The obtained outcomes are comparable to the state-of-the-art systems and achieved competitive results to solve the glaucoma eye disease problems for complex glaucoma eye disease cases.

**Figure 10:**
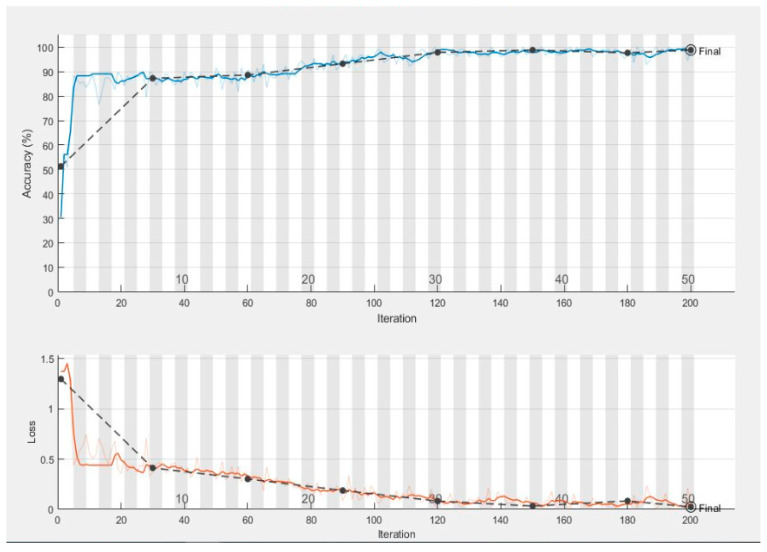
Graphical Representation of complete training process.

In Figure 10 the complete training process is represented graphically. The smooth line shows the training and the dotted line shows the validation of the dataset. In the end, we compared the results of our proposed model with the stat of the art techniques for glaucoma eye disease problem.

Table 6 shows the results of the state-of-the-art systems for the classification of advanced glaucoma, early glaucoma, moderate glaucoma, and normal glaucoma categories of glaucoma eye disease in recent years. As shown in Table 6, the proposed ML-DCNN algorithm gets better statistical values when compared to the other methods for the recognition of eye disease. The reason is that the ML-DCNN glaucoma model works in two parts one for glaucoma detection and the other one is glaucoma classification.

## 5. Conclusions

In this paper, the advanced machine deep-learning technique is used on retinal fundus images to diagnose glaucoma affected and normal images. To develop a multi-level deep convolutional neural network (ML-DCNN) for glaucoma detection and classification the CNN framework is implemented on 1338 images to extract features through a multilayer from raw pixel images. The ML-DCNN model is applied in two ways: one for detection of glaucoma as detection-net CNN (DN-CNN); and two for classification of glaucoma, known as classification-net (CN-CNN), into four categories. To evaluate the performance of the ML-DCNN model, the Specificity (SP), Sensitivity (SE), Accuracy (ACC) and Precision (PRC) statistical measures are used, and an average SE of 97.04%, SP of 98.99%, ACC of 99.39%, and PRC of 98.2% are achieved. The proposed ML-DCNN glaucoma model obtained different statistical values for advanced glaucoma, early glaucoma, moderate glaucoma, and normal glaucoma categories. The obtained outcomes are comparable to the state-of-the-art systems and achieved competitive results to solve the glaucoma eye disease problems for complex glaucoma eye disease cases. The proposed ML-DCNN method performs in a significant way, but in the future this model will be used for other eye diseases.

## Figures and Tables

**Table 1:** Description of Layers of Detection-Net Convolutional Neural Network (DN-CNN).

CNN Layers Description with Learning Rate = 0.001 and Epochs = 60		
Sr. No	Layers Description	Parameters
1	Image Input Layer	256 × 256
2	convolution2dLayer	256 × 256, 3 × 3, K = 8, stride = 1
3	Batch normalization Layer	3 × 3, stride = 1
4	Relu Layer	3 × 3, stride = 1
5	maxPooling2dLayer	3 × 3, stride = 1
6	convolution2dLayer	128 × 128, 3 × 3, k = 16, stride = 1
7	Batch normalization Layer	3 × 3, stride = 1
8	Relu Layer	3 × 3, stride = 1
9	maxPooling2dLayer	3 × 3, stride = 1
10	convolution2dLayer	64 × 64, 3 × 3, k = 32, stride = 1
11	Batch normalization Layer	3 × 3, stride = 1
12	Relu Layer	3 × 3, stride = 1
13	maxPooling2dLayer	3 × 3, stride = 1
14	Dropout Layer	Dropout = 0.7
15	Fully Connected Layer	2 Classes
16	Softmax Layer	2 Classes
17	classification Layer	2 Classes

**Table 2:** Description of Layers of Classification-Net CNN (CN-CNN).

	CNN Layers Description with Learning Rate = 0.001 and Epochs = 30	
Sr. No	Layers Description	Parameters
1	Image Input Layer	256 × 256
2	convolution2dLayer	256 × 256, 3 × 3, K = 8, stride = 1
3	maxPooling2dLayer	3 × 3, stride=1
4	convolution2dLayer	126 × 126, 3 × 3, k = 16, stride = 1
5	maxPooling2dLayer	3 × 3, stride = 1
6	convolution2dLayer	61 × 61, 3 × 3, k = 32, stride = 1
7	maxPooling2dLayer	3 × 3, stride = 1
8	Fully Connected Layer	3 Classes
9	SoftMax Layer	3 Classes
10	classification Layer	3 Classes

**Table 3:** Distribution of images in the different Groups (Classes) and Subsets.

Group	Total	Train	Validation	Test
Advanced Glaucoma	80	50	10	20
Early Glaucoma	120	75	15	30
Moderate Glaucoma	80	50	10	20
Normal	1058	628	200	230
**Total**	**1338**	**803**	**235**	**300**

**Table 4:** Computed Values (%) of Statistical Measures on Testing Dataset.

Detection of Glaucoma Eye Disease					
No	Category	Sensitivity (%)	Specificity (%)	Accuracy (%)	Precision (%)
1	Advanced Glaucoma	100.0	99.28	99.32	90.0
2	Early Glaucoma	93.54	99.62	98.9	96.6
3	Moderate Glaucoma	100.0	100.0	100.0	100.0
4	Normal	99.13	98.52	98.9	99.05
**Average**		**98.16**	**99.35**	**99.28**	**96.41**

**Table 5:** Computed Values (%) of Statistical Measures on the Local Retinal Glaucoma Image Dataset.

Detection of Glaucoma Eye Disease					
No	Category	Sensitivity (%)	Specificity (%)	Accuracy (%)	Precision (%)
1	Advanced Glaucoma	96.25	99.75	99.54	96.25
2	Early Glaucoma	92.21	99.75	99.02	97.5
3	Moderate Glaucoma	100.0	100.0	100.0	100.0
4	Normal	99.71	96.47	99.02	99.05
**Average**		**97.04**	**98.99**	**99.39**	**98.2**

**Table 6:** Comparison of Accuracy value (%) of the proposed technique with the stat of the art techniques.

Cited	Comparison of Detection of Glaucoma Eye Disease		
	Methodologies	Accuracy (%)	Year
[17]	CNN	83.00	2015
[25]	Feed-Forward neural network	92.00	2016
[19]	Support Vector Machine	87.00	2016
[21]	CNN	90.00	2016
[1]	Glaucoma-Deep (CNN, DBN d, Softmax)	99.0	2017
[10]	Semi-Supervised transfer learning for CNN)	92.4	2019
[11]	OCT Probability map using CNN	95.7	2019
[12]	AG-CNN	95.3	2019
Proposed ML-DCNN (Advanced, Early, Moderate and Normal Glaucoma)		99.39	2020
